# Longitudinal study of hearing preservation and electrocochleography after cochlear implantation in adults

**DOI:** 10.1016/j.bjorl.2025.101584

**Published:** 2025-03-26

**Authors:** Marianne Schleich, John J. Galvin, Fabrice Micaletti, David Bakhos

**Affiliations:** a University Hospital of Tours, Department of Otolaryngology – Head and Neck Surgery, Tours, France; b House Institute Foundation, Los Angeles, California, United States of America; c University Hospital of Tours, Tours, France

**Keywords:** Electrocochleography, ECOG, Residual hearing, Cochlear implant, Hearing preservation

## Abstract

**Objectives:**

Electrocochleography (ECOG) is a composite objective measure of the cochlea and the cochlear nerve responses. The objectives of this study were to investigate the evolution of ECOG after cochlear implantation and compare them with audiometric thresholds over a 12-month period.

**Methods:**

A prospective monocentric study was conducted in Advanced Bionics cochlear implant recipients. Audiometric thresholds were recorded preoperatively and at 1-, 3-, 6-, and 12-months post cochlear implantation; ECOGs were recorded perioperatively after the electrode array insertion and at 1-, 3-, 6-, and 12-months post cochlear implantation. Cochlear Microphonic (CM) responses were measured by an apical electrode of the cochlear implant. Postoperative behavioral and ECOG thresholds were compared using Pearson correlation analyses.

**Results:**

A total of 17 patients were included. At 1-year, residual hearing (≤90 dB HL at 500 Hz) was preserved for 4 out of 17 patients. Significant correlations were observed between perioperative ECOG and preoperative audiometric thresholds (*p* = 0.008), and between ECOG and audiometric thresholds at 3-months postoperative (*p* = 0.002).

**Conclusion:**

ECOG can reflect changes in cochlear responses during and after implantation and may help to predict deterioration of residual hearing.

**Level of evidence:**

Level 3.

## Highlights

• Residual hearing significantly deteriorates after cochlear implantation.
• 23.5% of the patients lose all residual hearing 1-year after cochlear implantation.
• Preoperative thresholds are good predictors of postoperative outcomes.
• ECOG correlates with postoperative audiometric thresholds in the short term.

## Introduction

Cochlear implantation can be indicated for patients with residual hearing in low frequencies.^1^ Residual hearing in implanted patients is beneficial, since it contributes to a higher quality of sound, better speech perception in noise,^2^, ^3^, ^4^ and greater music recognition.^5^, ^6^ However, the literature shows that residual hearing tends to deteriorate with time in implanted patients. A systematic review by Talbot and Hartley^7^ showed that out of 253 implanted patients with residual hearing, 13% lost their residual hearing completely, and 24% had a loss of >20 dB.

Previous studies have identified factors associated with deterioration of residual hearing after cochlear implantation. Intrinsic patient factors such as age, sex and etiology of deafness may impact the evolution of post-implantation residual hearing.^8^ Early residual hearing loss has been noted due to surgical trauma.^9^ Changes in electrode size,^10^, ^11^ flexibility^12^ and surgical insertion technique^13^, ^14^, ^15^, ^16^ have reduced the risk of intraoperative trauma. Finally, there are later impairments to residual hearing due to excitotoxicity^17^, ^18^ and the immune response to the foreign body in the cochlea.^19^, ^20^

Subjective audiometry is the reference test for post-implantation follow-up for acoustic hearing preservation. However, audiometry has limitations, particularly for children, in whom it can be difficult to reliably measure behavioral thresholds. Moreover, the audiogram alone may not be a sufficient measure of deterioration or loss of residual hearing because it cannot determine the origin of the lesion (cochlear or nerve damage).

Electrocochleography (ECOG) is a composite objective measure of the cochlea and the cochlear nerve responses. The response is recorded in the form of several potentials: the pre-synaptic Cochlear Microphonic (CM) corresponds to the Outer Hair Cell (OHC) micromechanical movements; the Summation Potential (SP) represents both the OHC and Inner Hair Cell (IHC) responses; the Compound Action Potential (CAP) and the Auditory Nerve Neurophonic (ANN) are post-synaptic potentials that represent the cochlear nerve response.^21^

There are few studies to date that compare post-implantation ECOG and audiometric thresholds over the long term.^22^, ^23^ The aims of this study were to investigate the evolution of ECOGs after cochlear implantation, and to compare ECOG with audiometric thresholds over a 12-month period. We hypothesized that ECOG would be predictive of postoperative audiometric thresholds in the short and long term, and thus would be a worthwhile objective measure of residual hearing over time.

## Methods

This was a prospective monocentric study in a French tertiary university hospital. We included patients who were candidates for cochlear implantation with the Advanced Bionics device between May 2021 and August 2022 who had residual hearing better than or equal to 90 dB at 500 Hz. Patients with auditory neuropathy and/or cochlear anatomical abnormalities were excluded. A total of 19 patients were enrolled in the study; two patients were later excluded due to insufficient data. Table 1 shows demographic information for the 17 patients who completed the study. This study was approved by the French data protection authority (Commission Nationale de l’Informatique et des Libertés; n°2021_061) and each patients signed a written consent.

**Table 1:** Patient characteristics.

Patient	Sex	Side of CI	Etiology of DFN	Time since hearing loss (Y)	Age at implantation (Y)
1	F	L	Unknown	21	49
2	M	R	Genetic	18	71
3	F	R	Unknown	15	66
4	F	R	Unknown	11	60
5	M	L	Unknown	10	83
6	M	L	Unknown	20	82
7	F	R	SSHL	1	41
8	M	R	Genetic	25	73
9	F	L	Unknown	20	83
10	M	R	Unknown	NC	64
11	M	R	Unknown	10	87
12	F	L	SSHL	10	71
13	F	L	Unknown	14	86
14	M	L	Genetic	10	62
15	F	R	SSHL	4	68
16	F	R	Unknown	20	36
17	F	R	Unknown	11	67

DFN, Deafness; CI, Cochlear Implant; F, Female; M, Male; R, Right; L, Left; Y, Years; SSHL, Sudden Sensorineural Hearing Loss.

**Fig. 1:**
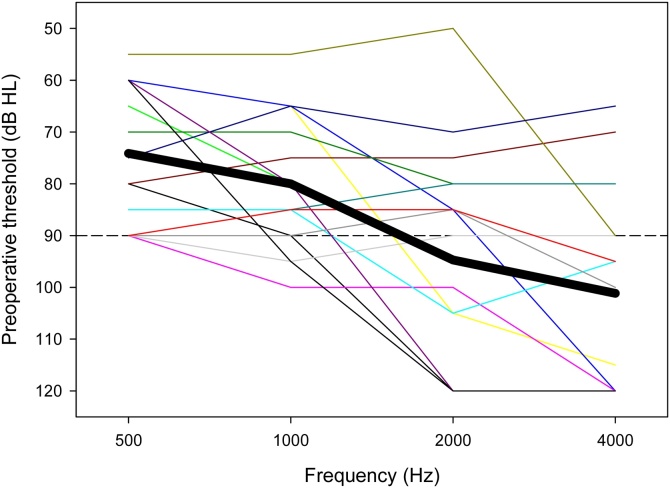
Preoperative behavioural thresholds. Each line represents the audiometric threshold of one patient. The bold line represents the mean threshold across the 17 patients.

For each patient, a CT scan of the temporal bone, an MRI of the internal ear, and pure-tone audiometry were performed as part of the preoperative work-up. Fig. 1 shows preoperative audiometric thresholds for all participants. ECOGs were collected during surgery, after the electrode array insertion. In the postoperative period, a CT scan of the temporal bone confirmed the position of the electrode. At 1-, 3-, 6-, and 12-months after surgery, ECOGs and audiometric thresholds at 500 Hz were measured again, within the limits of patient’s availability.

### ECOG measurement technique

An ER-3A insert (Etymotic Research, Inc. 61 Martin Lane, Elk Grove Village, IL) and an ER-7 microphone (Etymotic Research, Inc. 61 Martin Lane, Elk Grove Village, IL) were placed in the ear canal of the implanted ear. The implant electrodes and the Naida CI Q90 sound processor were used to measure ECOGs using the manufacturer’s equipment (Advanced Bionics’ Active Insertion Monitoring system). Pure-tone acoustic stimuli at 500-, 1000-, 2000-, and 4000-Hz, 115 dB SPL, and 50 ms in duration were used to measure ECOG responses. For each subject the most apical electrode was used as the recording electrode. However, ECOG responses were unreliable at 1000-, 2000-, and 4000-Hz, most likely due to the limited acoustic hearing in the present participants. As such, ECOG responses are reported only for 500 Hz, consistent with Tejani et al.^23^ The CM response was compared to the noise floor; the response was considered significant if it was 3 times above the noise floor.

### Statistical analysis

All statistical analyses were performed using SPSS software (version 20; Armonk, NY). Characteristics of the participants, audiometric thresholds and ECOG responses after implantation were described according to their nature by sample size and percentages or by mean and standard deviation. A Kaplan-Meier survival analysis was performed on the behavioral 500-Hz threshold data, with threshold > 90 dB HL as the outcome.

Linear Mixed Model (LMM) analysis was performed on the behavioral threshold and ECOG data, with time (preoperative, 1-month postoperative, 3-months postoperative, 6-months postoperative, 12-months postoperative) as the fixed factor and participant as the random factor; age and duration of hearing loss were co-variables. Because age at test and age at CI were strongly correlated (*r* = 0.99, *p* < 0.001), these variables were collapsed into a single factor (“age”) for subsequent analysis using Principal Components Analysis (PCA).

Preoperative behavioral thresholds at 500 Hz were compared to postoperative thresholds at 1-, 3-, 6-, and 12-months. Perioperative ECOGs at 500-Hz were compared to postoperative ECOGs at 1-, 3-, 6-, and 12-months. Postoperative behavioral and ECOG thresholds were compared at 1-, 3-, 6-, and 12-months postoperative. Pearson correlations were used for all comparisons.

For all statistical tests, significance was *p* < 0.05.

## Results

### Audiometric thresholds evolution

**Fig. 2:**
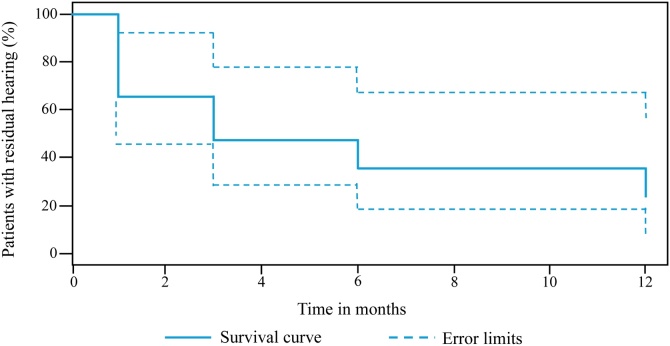
Percentage of patients with residual acoustic hearing ≤90 dB HL as a function of time. Time 0, Preoperative thresholds. Time >1, Postoperative thresholds.

All patients had audiometric thresholds ≤90 dB HL at 500-Hz prior to surgery. Mean thresholds at 500-Hz were 74 ± 13 dB HL preoperatively, 101 ± 15 dB HL, 100 ± 16 dB HL, 102 ± 15 dB HL, and 107 ± 16 dB HL, at 1-, 3-, 6-, and 12-months postoperatively, respectively. Residual hearing tended to decline, especially during the first month after cochlear implantation. Fig. 2 shows the results of the Kaplan-Meier survival analysis; the percentage of patients where the audiometric threshold at 500-Hz were ≤90 dB HL is shown as a function of time. After 12-months, only 4 out of 17 patients had residual hearing ≤ 90 dB HL at 500-Hz.

LMM analysis was performed on the behavioral threshold data, with time (preoperative, 1-, 3-, 6-, and 12-months postoperative) as the fixed factor, participant as the random factor, and age and duration of hearing loss as co-variables. Results showed significant effects for time [F(4,40.3) = 5.1, *p* = 0.002] and age [F(1,11.8) = 6.3, *p* = 0.027], but not for duration of hearing loss [F(1,11.8) = 2.6, *p* = 0.134]; there were no significant interactions among time, age, and duration of hearing loss. Post-hoc Bonferroni comparisons showed that preoperative thresholds were significantly lower (better) than at 1-, 3-, 6-, or 12-months postoperative, and significantly lower at 6-months postoperative than at 12-months postoperative (*p* < 0.05 in all cases).

**Fig. 3:**
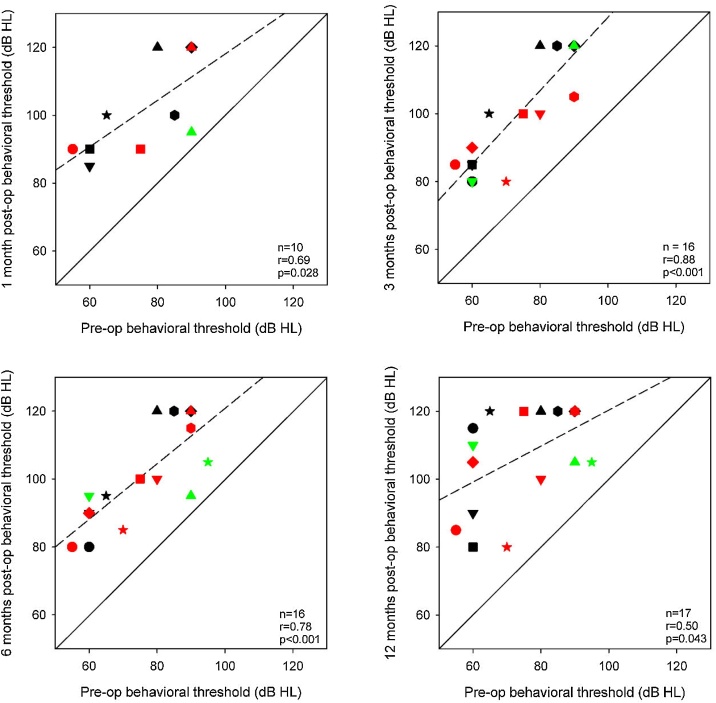
Postoperative 500-Hz behavioral thresholds at 1-, 3-, 6-, and 12-months as a function of preoperative thresholds. The different symbols and colors represent individual patient data and are consistent across panels. The solid diagonal line represents unity; values above the line indicate higher (poorer) postoperative thresholds relative to preoperative thresholds. The dashed line shows the regression through the data; r and p-values are shown at the bottom right of each panel. Note that data were not available for all patients at all time points; hence there is missing data for some patients in some panels.

Fig. 3 shows behavioral thresholds at 500-Hz at 1-, 3-, 6-, and 12-months postoperative as a function of preoperative thresholds. Values above the solid diagonal line indicate that postoperative thresholds are higher than preoperative thresholds. The dashed line shows the linear regression through the data. Note that behavioral data was not available for all participants at all time points. Significant correlations were observed at all time points, suggesting that preoperative thresholds were a good predictor of postoperative thresholds. Preoperative thresholds were ≤90 dB HL for all participants. At 12-months postoperative, thresholds for all but 4 participants were >90 dB HL.

### ECOG evolution

Mean ECOG thresholds at 500-Hz were 95 ± 17 dB during surgery (after cochlear implant insertion in the round window), 97 ± 13, 95 ± 14, 102 ± 10, and 101 ± 14 dB, at 1-, 3-, 6-, and 12-months postoperatively, respectively. LMM analysis was also performed on the ECOG threshold data, with time as the fixed factor, participant as the random factor, and age and duration of hearing loss as co-variables. Results showed no significant effects for time [F(4,38) = 1.0, *p* = 0.410], age [F(1,14.3) = 3.1, *p* = 0.099], or duration of hearing loss [F(1,12.5) = 1.8, *p* = 0.204]; a significant interaction was observed only between age and duration of hearing loss [F(1,14.2) = 5.6, *p* = 0.033].

**Fig. 4:**
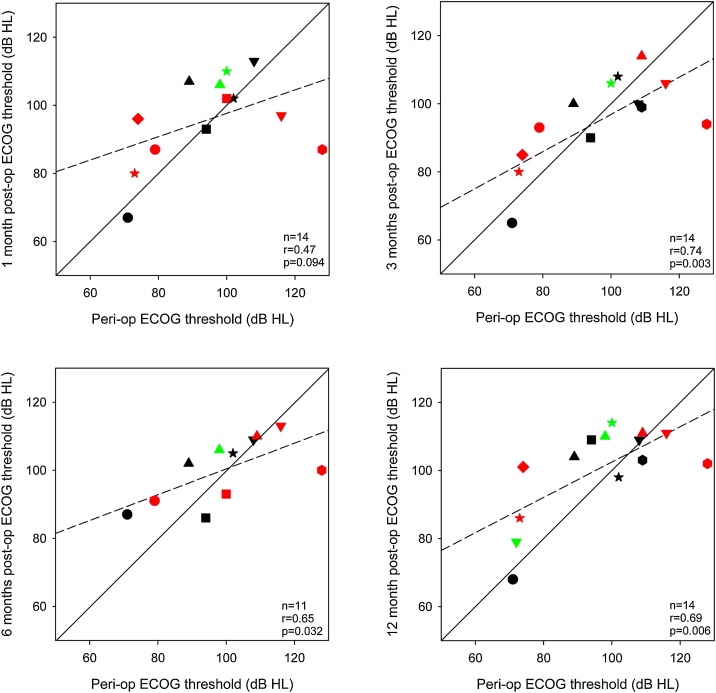
Postoperative ECOG thresholds at 1-, 3-, 6-, and 12-months as a function of peri-operative thresholds. The different symbols and colors represent individual patient data and are consistent across panels. The solid diagonal line represents unity; values above the line indicate higher (poorer) postoperative thresholds relative to peri-operative thresholds. The dashed line shows the regression through the data; r and p-values are shown at the bottom right of each panel. Note that data were not available for all patients at all time points; hence there is missing data for some patients in some panels.

Similar to Fig. 3, Fig. 4 shows postoperative ECOG thresholds at 500-Hz at 1-, 3-, 6-, and 12-months as a function of perioperative thresholds (after the electrode array insertion). Significant correlations were observed at 3-, 6-, and 12-months postoperative, but not at 1-month postoperative. Perioperative thresholds were ≤90 dB HL for only 5 participants. At 12-months postoperative, thresholds for all but 3 participants were >90 dB HL. If the threshold cut-off is changed to 100 dB HL, perioperative thresholds were ≤100 dB HL for 10 participants; at 12-months postoperative, thresholds for all but 4 participants were >100 dB HL.

### Relationship between ECOG and audiometric thresholds

**Fig. 5:**
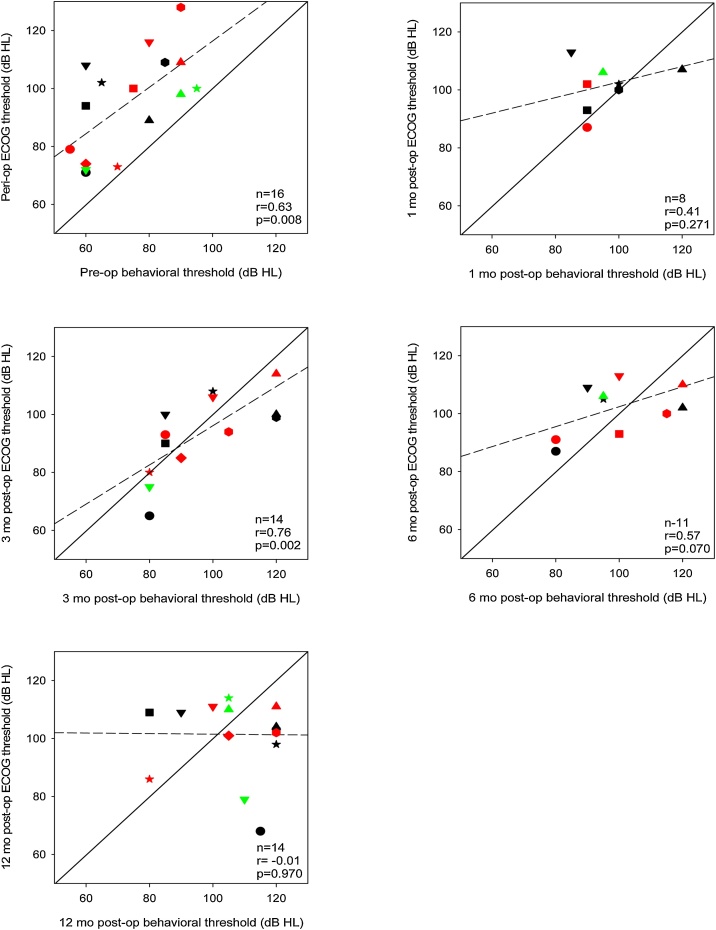
ECOG thresholds as a function of behavioral thresholds. Data are shown for perioperative ECOGs vs. preoperative behavioral thresholds, and for ECOG vs. behavioral thresholds at 1-, 3-, 6-, and 12-months postoperative. The different symbols and colors represent individual patient data and are consistent across panels. The solid diagonal line represents unity; values above the line indicate higher (poorer) ECOG thresholds compared to behavioral thresholds. The dashed line shows the regression through the data; r and p-values are shown at the bottom right of each panel. Note that data were not available for all patients at all time points; hence there is missing data for some patients in some panels.

Fig. 5 shows 500-Hz ECOG thresholds as a function of 500-Hz behavioral thresholds before surgery, during surgery (after the electrode array insertion), and at 1-, 3-, 6-, and 12-months postoperative. Significant correlations were observed only between perioperative ECOG and preoperative behavioral thresholds (*p* = 0.008), and between ECOG and behavioral thresholds at 3-months postoperative (*p* =  0.002). By 12-months postoperative, there was no relation whatsoever between ECOG and behavioral thresholds.

## Discussion

### Residual hearing

In this study, audiometric thresholds significantly increased during the first year after cochlear implantation, with most patients (13/17) completely losing their residual hearing. As shown in Fig. 3, better thresholds before surgery were strongly predictive of thresholds after surgery. Previous studies have demonstrated significant preservation of residual hearing after cochlear implant surgery.^2^, ^6^, ^7^, ^24^, ^25^, ^26^, ^27^, ^28^ In those studies, inclusion criteria were 500-Hz thresholds ≤65 dB HL. In the present study, patients had less acoustic hearing before implantation, with a mean preoperative 500-Hz threshold of 73 dB HL, with 90 dB HL thresholds for 5/17 patients. Previous studies had shorter follow-ups of 1- and 3-months,^6^, ^25^, ^26^, ^27^ versus the 1- to 12-month follow-up in the present study.

Preserving post-implantation residual hearing over the long term remains a challenge. There are different approaches to preserve residual hearing. Surgical trauma to the cochlea can be reduced by using new technologies, such as robotics-assisted cochlear implantation.^16^, ^29^ Electrode array insertions performed by robotics-assisted system showed significantly lower insertion forces and less variability than observed with manual insertions. Decreasing the auto-immune response against the foreign body either by dexamethasone^20^, ^30^ or by a protective film on the electrode array^31^, ^32^ may help to preserve residual hearing. Dexamethasone diffusion in the inner ear via drug-eluting electrodes may reduce the local inflammatory response by reducing the macrophages’ action and thereby minimizing cochlear fibrosis. Biomaterial coatings of the electrode array may limit friction and forces during electrode insertion, thereby reducing inflammation and secondary fibrosis in the cochlea. ECOGs can be used for intraoperative monitoring of electrode insertion depth through the round window. Previous studies have shown that ECOG amplitudes during insertion are predictive of postoperative residual hearing preservation.^33^, ^34^, ^35^, ^36^ However, it is still unclear how ECOGs may be used to guide electrode insertion to better preserve residual hearing.

When hearing preservation is possible, “hybrid” CI signal processors can provide both low-frequency acoustic stimulation via hearing aid and higher-frequency electric stimulation via CI (combined “Electro-Acoustic Stimulation”, or EAS). In general, EAS patients have a maximum hearing loss of 65 dB between 125 and 750 Hz.^11^ The benefit of EAS relies on preservation of residual hearing over the long term.

## ECOG

Previous studies have reported significant associations between behavioral and postoperative ECOG thresholds.^22^, ^37^, ^38^, ^39^, ^40^, ^41^, ^42^ Most previous studies compared ECOG and audiometric thresholds at only one or two postoperative test points;^37^, ^38^, ^41^, ^42^, ^43^ there are few studies with long-term monitoring of ECOG evolution.^22^, ^23^ In the present study, follow-up measures were conducted out to 12-months postoperative. ECOG measures during surgery and preoperative behavioral thresholds at 500 Hz were significantly correlated as well as at 3-months postoperative; no correlations were observed at later follow-ups.

## Conclusion

In the present study, while low-frequency residual hearing could sometimes be preserved at 1- or 3-months post-implantation, most patients (13/17) lost their residual hearing by 12-months post-implantation. The correlations observed between 500 Hz ECOG and behavioral thresholds suggest that ECOG can predict changes in cochlear responses during and after implantation.

## Authors’ contributions

Study conception, Material preparation, and analysis were performed by Marianne Schleich, John Galvin and David Bakhos. Data was collected by Fabrice Micaletti and Marianne Schleich. The first draft of the manuscript was written by Marianne Schleich and all authors commented on previous versions of the manuscript. All authors read and approved the final manuscript.

## Statements and declarations

This manuscript is not under consideration elsewhere.

Ethical approval: This study was approved by the French data protection authority (CNIL; nº 2021_061) and the local ethics committee in human research (nº 2021_029). The procedures used in this study adhere to the tenets of the Declaration of Helsinki.

Informed consent: Informed consent was obtained from all individual participants included in the study.

Data availability statement: The data that support the findings of this study are available from the corresponding author upon reasonable request

## Funding

This study was not supported by any funding.

## Declaration of competing interest

The authors declare no conflicts of interest.
